# Quantitative, Dynamic ^18^F-FDG PET/CT in Monitoring of Smoldering Myeloma: A Case Report

**DOI:** 10.3390/diagnostics11040649

**Published:** 2021-04-03

**Authors:** Christos Sachpekidis, Matthias Türk, Antonia Dimitrakopoulou-Strauss

**Affiliations:** Clinical Cooperation Unit Nuclear Medicine, German Cancer Research Center, Heidelberg 69120, Germany; matthias.tuerk@gmx.de (M.T.); a.dimitrakopoulou-strauss@dkfz.de (A.D.-S.)

**Keywords:** smoldering myeloma, multiple myeloma, quantitative, dynamic ^18^F-FDG PET/CT, autologous stem cell transplantation

## Abstract

We report on a 52-year-old patient with an initial diagnosis of smoldering myeloma (SMM), who was monitored by means of dynamic and static positron emission tomography/computed tomography (PET/CT) with the radiotracer ^1^⁸F-fluorodeoxyglucose (^18^F-FDG). Baseline PET/CT revealed no pathological signs. Six months later, a transition to symptomatic, multiple myeloma (MM) was diagnosed. The transition was not accompanied by focal, hypermetabolic lesions on PET/CT. However, a diffusely increased ^18^F-FDG uptake in the bone marrow, accompanied by a marked increase of semi-quantitative (standardized uptake value, SUV) and quantitative, pharmacokinetic ^18^F-FDG parameters, was demonstrated. After successful treatment, including tandem autologous transplantation, the diffuse uptake in the bone marrow as well as the semi-quantitative and quantitative parameters showed a marked remission. This response was also confirmed by the clinical follow-up of the patient. These findings suggest that in MM a diffuse ^18^F-FDG uptake in the bone marrow may indeed reflect an actual bone marrow infiltration by plasma cells. Moreover, SUV values and kinetic parameters, not only from myeloma lesions but also from random bone marrow samples, may be used for MM monitoring. This could be particularly helpful in the follow-up of myeloma patients negative for ^18^F-FDG-avid focal lesions.

## 1. Introduction

Multiple myeloma (MM) is a neoplastic plasma cell disorder, characterized by the uncontrolled, clonal proliferation of plasma cells in the bone marrow. It is the second most common hematologic malignancy after non-Hodgkin lymphoma and accounts for approximately 1% of neoplastic diseases [1]. MM is almost always preceded by a premalignant precursor condition (monoclonal gammopathy of undetermined significance, MGUS), which then develops into asymptomatic or smoldering myeloma (SMM) and, finally, into symptomatic disease [2,3]. SMM represents a highly heterogeneous entity with a progression risk to MM of 10% per year during the first five years after diagnosis [4]. Different risk stratification models for progression of SMM to MM have been developed, the most popular being the Mayo Clinic model, which utilizes M-protein, bone marrow plasma cell infiltration and the ratio of serum free light chain, and the Spanish model, which uses flow-cytometry to define the proportion of aberrant plasma cells in the marrow and the presence of immunoparesis [5,6]. The identification of those SMM patients who have a high risk of progression to active, symptomatic disease is of utmost clinical importance, since these patients would benefit from early treatment commencement [7,8]. In this context, in 2014, the definition of MM was revised by the International Myeloma Working group (IMWG), including the subset of SMM patients with an 80% two-year risk of progression to symptomatic MM, based on findings from bone marrow biopsy, serum free light chain and magnetic resonance imaging (MRI) [9].

The role of positron emission tomography/computed tomography (PET/CT) with the radiotracer ^1^⁸F-fluorodeoxyglucose (^18^F-FDG) in multiple myeloma (MM) has been markedly upgraded in recent years. A steadily increasing amount of literature has highlighted the value of the imaging modality in diagnosis, prognosis and treatment response evaluation of the disease [10,11,12,13,14,15]. Proof of the established role of PET/CT in MM management, is its inclusion in the latest updated criteria for the diagnosis of the disease by IMWG. In particular, the detection of one or more osteolytic lesions on CT or PET/CT fulfills the criteria of bone disease and, therefore, of symptomatic MM requiring treatment [10]. Although still limited, the first results of the application of ^18^F-FDG PET/CT in asymptomatic SMM have been promising, reflecting the potential role of the modality in predicting the risk of progression to symptomatic disease [16,17]. 

On the other hand, in myeloma—more than in other malignancies—issues on the evaluation of ^18^F-FDG PET/CT exist. This is mainly attributed to the different patterns of bone marrow involvement in the disease, which results in poor inter-observer reproducibility in scan interpretation [18]. In an attempt to standardize the interpretation of ^18^F-FDG PET/CT scans in MM, several efforts have been undertaken, making use of visual [18,19] as well as semi-quantitative and quantitative approaches [20,21,22,23,24]. However, none of these methods have been yet extensively applied in clinical practice.

We herein report on a patient with an initial diagnosis of SMM who was monitored throughout the physical history of the disease by means of quantitative, dynamic ^18^F-FDG PET/CT.

## 2. Case Report

**Figure 1:**
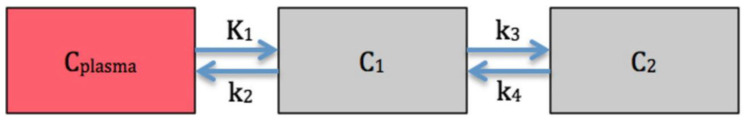
Schematic representation of the two-tissue compartment model applied for ^18^F-FDG. K_1_, k_2_, k_3_ and k_4_ are rate constants (1/min) and describe the directional exchanges between the three compartments. C_plasma_ represents the vascular compartment, C_1_ represents the free and non-specifically bound tracer in tissue (non-displaceable compartment) and C_2_ represents the specifically bound (phosphorylated) tracer in tissue. K_1_ reflects the carrier-mediated transport of ^18^F-FDG from plasma to tissue and k_2_ reflects the transport of the radiopharmaceutical back from tissue to plasma, while k_3_ represents the phosphorylation rate and k_4_ the dephosphorylation rate of the glucose analogue.

A 52-year-old male patient with a diagnosis of SMM of IgG-lambda type (initial bone marrow plasma cell infiltration rate 10%) was referred to the nuclear medicine department for staging purposes. The patient underwent both dynamic and static PET/CT (Biograph mCT, S128, Siemens Co., Erlangen, Germany) with ^18^F-FDG. In particular, after an intravenous bolus administration of ^18^F-FDG, dynamic PET/CT was performed over the lower abdomen and pelvis for 60 min using a 24-frame protocol (10 frames of 30 s, 5 frames of 60 s, 5 frames of 120 s and 4 frames of 600 s). After the end of the dynamic acquisition, whole body, static imaging from the head to the feet was performed with an image duration of 2 min per bed position for the emission scans. Data analysis of PET/CT consisted of the conventional visual (qualitative) and semi-quantitative (standardized uptake value, SUV) evaluation, as well as the quantitative analysis of the dynamic ^18^F-FDG PET/CT data, which was based on two-tissue compartment modeling (Figure 1) [25,26,27,28] and fractal analysis [29].

**Figure 2:**
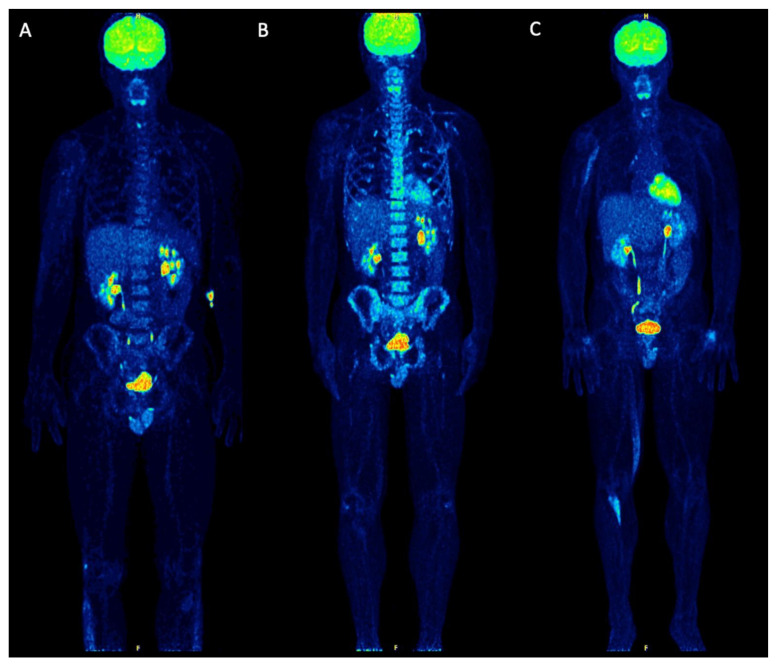
Maximum intensity projection (MIP) ^18^F-FDG PET/CT images upon SMM diagnosis (**A**), at transition to symptomatic MM (**B**) and after therapeutic intervention (**C**). Baseline PET/CT upon asymptomatic SMM showed no pathological findings. A discretely increased, diffuse tracer uptake in the bone marrow (≤liver uptake) is observed. The foci of increased ^18^F-FDG uptake in the lower abdomen/pelvis correspond to physiological urinary tracer activity in the ureters, while the focal ^18^F-FDG accumulation in the right knee joint most likely represents an inflammatory process. Semi-quantitative calculations revealed a SUV_mean_ of the iliac bone of 2.2 (SUV_max_ 3.3). According to the quantitative, pharmacokinetic analysis, the influx of ^18^F-FDG in the bone marrow was 0.01 (1/min) (**A**). The first follow-up PET/CT scan at the time of symptomatic myeloma transition (6 months later) revealed an intense, diffuse ^18^F-FDG uptake in the bone marrow of the axial skeleton without any focal lesions. SUV_mean_ of the iliac bone increased to 4.0 (SUV_max_ 6.3), while the tracer influx increased to 0.03 (1/min) (**B**). Two months after successful treatment, which involved tandem HDT and ASCT, the patient underwent a second follow-up PET/CT scan. This demonstrated a pronounced remission of the diffuse bone marrow uptake and a reduction of the respective SUV values (SUV_mean_ 1.4, SUV_max_ 1.6) and influx (0.01 (1/min)) of ^18^F-FDG. The elongated uptake in the right arm, right thigh and lower leg correspond to muscular activity. Physiological urinary tracer activity is observed in the ureters (**C**).

**Figure 3:**
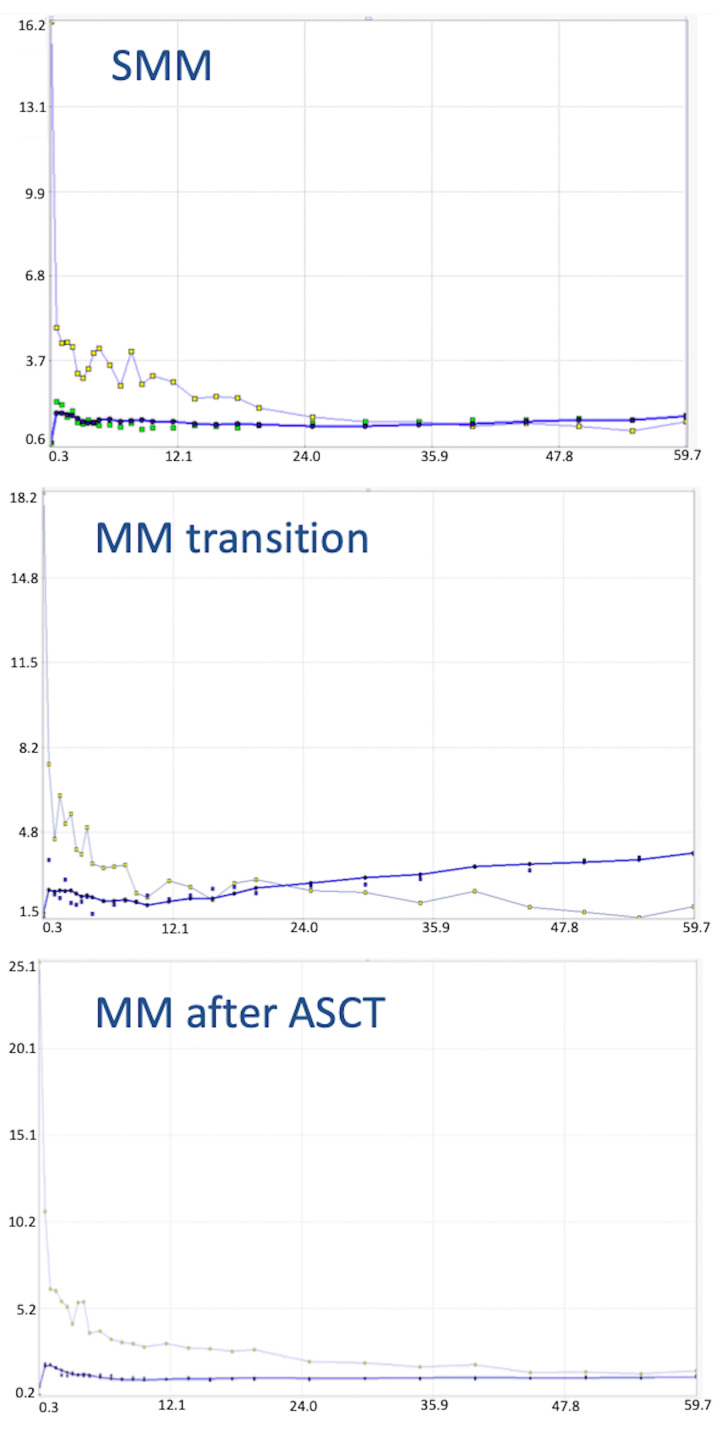
Time activity curves (TACs) depicting ^18^F-FDG concentration during the 60 min of dynamic PET acquisition upon SMM diagnosis (upper row), at transition to symptomatic myeloma (middle row) and after therapy with tandem HDT and ASCT (lower row). The curves are derived from the bone marrow of the iliac bone (thick blue curve with green dots) and from the common iliac artery (thin blue curve with golden dots). The TAC at baseline imaging shows a relatively stable tracer concentration over time. The transition from asymptomatic SMM to symptomatic MM is accompanied by a change in the respective TAC of the radiotracer, showing a steadily increasing accumulation in the bone marrow compared to the definitely lower tracer concentration upon SMM. After the therapeutic intervention, we notice a decrease of the ^18^F-FDG concentration in the bone marrow, which is also reflected in the slope of the curve.

Baseline PET/CT at SMM diagnosis revealed no pathological findings suggestive of myeloma. Merely, a discretely increased, diffuse tracer uptake in the bone marrow was noticed, which was, however, not higher than liver uptake (Figure 2A). Six months later, a transition from asymptomatic SMM to symptomatic MM was diagnosed, after a pathological fracture of the right humerus—treated with surgery and radiotherapy—accompanied by respective increases of the M-protein (from 3.1 g/dL to 6.1 g/dL), and the lambda light chains in serum (from 135 mg/L to 306 mg/L) and urine (from unmeasurable levels to 20.6 mg/24 h). The patient was re-assessed with dynamic and static PET/CT, which demonstrated no focal hypermetabolic lesions. However, a new, intense, diffuse ^18^F-FDG uptake in the bone marrow of the axial skeleton was now delineated (Figure 2B). With regard to the semi-quantitative PET/CT parameters, SUV_mean_ and SUV_max_ of the iliac bone increased by 82% and 91%, respectively, in comparison to the baseline scan. Similar changes were observed in the pharmacokinetic parameters derived from dynamic PET/CT: the regional blood volume (V_B_) increased by 150%, the tracer influx (K_i_) increased by 200%, and fractal dimension (FD) also increased by 16% (Table 1; Figure 3). The patient was treated with bortezomib-based induction therapy, followed by tandem high-dose chemotherapy (HDT) and autologous stem cell transplantation (ASCT). Two months after therapy, a third PET/CT demonstrated a pronounced remission of the diffuse bone marrow uptake (Figure 2C), accompanied by a marked decrease of the respective semi-quantitative and quantitative parameters to levels similar to or even lower than those of baseline PET/CT (Table 1; Figure 3). These findings were in line with respective changes of the M-protein (decrease to 1.1 g/dL), and the lambda light chains in serum (decrease to 24 mg/L) and urine (decrease to unmeasurable levels). The patient, furthermore, received maintenance therapy with lenalidomide. At last contact, he had not shown any disease progression, having reached a progression-free survival (PFS) of 74 months.

## 3. Discussion

^18^F-FDG PET/CT is regarded as a reliable outcome predictor and an elective imaging technique for treatment response evaluation of MM due to its ability in differentiating active from inactive sites of the disease [30]. Three independent, easily attainable with routine PET/CT parameters have been recognized to adversely affect both PFS and overall survival (OS). In particular, the presence of more than three focal ^18^F-FDG-avid lesions, a SUV_max_ > 4.2 of the lesions and the presence of extramedullary disease (EMD) are associated with an adverse outcome [12,31]. Moreover, the complete remission of the ^18^F-FDG-avid lesions after therapy has been shown to confer superior PFS and OS; contrarily, the persistence of pathologic findings on PET/CT after treatment is associated with a worse prognosis [12,13,14,31,32].

Thus far, the vast majority of PET/CT studies in MM were restricted either to descriptive analyses, mainly through the identification of focal, hypermetabolic lesions, and/or semi-quantitative analyses of parameters derived from static imaging of focal lesions. Little light, however, has been shed on the interpretation and prognostic value of the diffuse bone marrow involvement—irrespective of the presence of focal lesions—on PET/CT. Moreover, the quantitative aspect of PET, which is feasible only after performance of dynamic scanning, has only been scarcely utilized, due to the routine application of conventional, static protocols.

In the present case, we monitored by means of dynamic and static PET/CT a patient with an initially asymptomatic SMM, who demonstrated a transition to symptomatic myeloma, and was subsequently successfully treated. The patient did not show any typical signs of myeloma involvement on PET/CT, i.e., focal, hypermetabolic lesions, at any phase during the course of the disease. However, at transition from SMM to symptomatic MM, a diffusely increased ^18^F-FDG uptake in the bone marrow was observed; this was accompanied by a marked increase of both the semi-quantitative (SUV values) and the quantitative, pharmacokinetic parameters, derived from bone marrow of the iliac crest. Importantly, after the successful therapeutic intervention, the diffuse uptake in the bone marrow as well as the semi-quantitative and quantitative parameters showed a pronounced remission. This response was also confirmed by the long-term, clinical follow-up of the patient.

Our findings suggest, firstly, that in untreated MM, a diffuse ^18^F-FDG uptake in the bone marrow may reflect an actual bone marrow infiltration by plasma cells. Particularly in patients suffering from SMM, the appearance of a diffuse hypermetabolic bone marrow pattern—regardless of the concurrent emergence of focal lesions—during the course of the entity may reflect transition to symptomatic disease and should, therefore, lead to further investigation. We are, indeed, aware of the several causes leading to a false-positive, diffuse, homogeneous, bone marrow ^18^F-FDG uptake on PET/CT, such as severe anemia, previous administration of granulocyte colony-stimulating factor (G-CSF), chemotherapy or erythropoietin [33]. However, in the present case, all potential causes of a false-positive bone marrow ^18^F-FDG uptake could be excluded from the patient’s history.

Secondly, SUV values not only from myeloma lesions—as consistently highlighted by previous studies—but also from random bone marrow samples, may be used to monitor disease transition and response to treatment. This could be particularly helpful in the follow-up of myeloma patients negative for ^18^F-FDG-avid focal lesions. The metabolic state of the bone marrow as evaluated by SUV calculations and/or in comparison to reference organs has recently been put into focus of MM research, rendering promising results as a potentially prognostic factor [22,34]. The present findings are in support of this direction.

Finally, the information acquired after the application of full dynamic PET/CT was in line with the respective qualitative (visual) and semi-quantitative (SUV) findings during the different phases of the disease. The dynamic ^18^F-FDG PET/CT protocol offers the unique advantages to investigate the tracer accumulation over time through generation of the respective time activity curves (TACs) as well as to extract pharmacokinetic indices that reflect dedicated parameters of the tracer’s metabolism, such as perfusion, transport or phosphorylation. This quantitative aspect is a major advantage of PET/CT, which is neglected when using conventional, static, whole-body protocols (usually 60 min post-injection) and descriptive analysis as the only diagnostic tool. In the present case, quantitative, dynamic PET/CT showed that the transition of asymptomatic SMM to symptomatic disease was accompanied by a marked increase of ^18^F-FDG accumulation in the bone marrow over time, compared to baseline PET/CT. Moreover, several quantitative ^18^F-FDG parameters, including the regional blood volume (V_B_), the tracer influx rate (K_i_), the carrier-mediated transport of the tracer from plasma to bone marrow (K_1_), the phosphorylation rate of ^18^F-FDG in the bone marrow (k_3_) as well as the degree of tracer heterogeneity—reflected by the parameter fractal dimension (FD)—showed a distinct increase. Contrarily, a pronounced decrease of the respective TAC and pharmacokinetic parameters were observed after the successful therapeutic intervention. These results are in line with previous findings of our group regarding the potential role of dynamic PET in MM prognosis and treatment response evaluation [22,35,36,37]. By complementing the information offered by conventional imaging with the multiparametric, pharmacokinetic data extracted by dynamic PET/CT, the diagnostic certainty of the reading physician could be enhanced, particularly in patients with ambiguous findings. Moreover, our understanding of the pathophysiology of the disease and its response to treatment can be improved.

Although these findings could suggest the wider usage of dynamic PET/CT in MM, more data, preferably derived from large prospective studies, are warranted to prove the potential benefit of the modality. Moreover, we note some practical considerations related to the possible implementation of dynamic PET/CT in clinical routine: firstly, it is more time-consuming than conventional PET/CT, since it requires in most cases a 60-min acquisition, followed by the conventional, static, whole-body PET/CT acquisition. This may lead to patient discomfort as well as to logistical issues in a nuclear medicine department. Furthermore, data interpretation is challenging, based on sophisticated software tools and application of the—rather complex—compartment modeling and fractal analysis. According to the previous, for the time being, qualitative and semi-quantitative analysis will remain the main evaluation tools of PET/CT in MM. However, dynamic PET/CT could be applied in selected cases, for example in the context of clinical trials in MM, adding significant quantitative information and reducing inter-observer variability. Moreover, the recent advent of new PET/CT scanners, which allow dynamic studies over several bed positions by using a continuous bed movement, will facilitate the use of dynamic PET protocols and reduce the whole acquisition time, making dynamic PET/CT an attractive and cost-effective approach in oncological imaging [38].

## 4. Conclusions

A patient with an initial diagnosis of asymptomatic SMM was monitored by means of dynamic and static ^18^F-FDG PET/CT during the course of the disease. Upon SMM diagnosis, the patient had no pathological signs on PET/CT. The transition from SMM to symptomatic MM was not accompanied by the typical signs of myeloma involvement on PET/CT, i.e., focal, hypermetabolic lesions. However, a diffusely increased ^18^F-FDG uptake in the bone marrow was observed, while at the same time, a marked increase of both semi-quantitative (SUV values) and quantitative, pharmacokinetic parameters was demonstrated. Following treatment, the diffuse uptake in the bone marrow as well as the semi-quantitative and quantitative parameters showed a pronounced remission. This response was also confirmed by the long-term, clinical follow-up of the patient. Altogether, the here-presented findings suggest, firstly, that in MM a diffuse ^18^F-FDG uptake in the bone marrow may reflect an actual bone marrow infiltration by plasma cells. Secondly, SUV values not only from myeloma lesions—as highlighted by previous studies—but also from random bone marrow samples, may be used for MM monitoring; this could be particularly helpful in the follow-up of myeloma patients negative for ^18^F-FDG-avid focal lesions. Finally, several pharmacokinetic parameters, derived from dynamic PET/CT, can be used to increase the diagnostic certainty and provide valuable information on dedicated parameters of the tracer’s metabolism.

## Figures and Tables

**Table 1:** Semi-quantitative and quantitative parameters of the bone marrow (iliac bone) derived from dynamic PET/CT at the three time points of scanning. The units of parameters fractional blood volume (V_B_), K_1_, k_3_ and influx (K_i_) are 1/min. SUV_mean_, SUV_max_ and fractal dimension (FD) have no unit.

Parameter	Baseline PET/CT (SMM)	First Follow-Up PET/CT (MM Transition)	Second Follow-Up PET/CT (MM after ASCT)
SUV_mean_	2.2	4.0	1.4
SUV_max_	3.3	6.3	1.6
V_B_	0.02	0.05	0.001
K_1_	0.31	0.39	0.18
k_3_	0.03	0.09	0.04
Influx (K_i_)	0.01	0.03	0.01
FD	1.09	1.26	1.05

## Data Availability

The authors confirm that the data supporting the findings of this case report are available within the article.
